# P‑Doped Carbon Nanotubes as Light-Absorbing Electron Donors in Photovoltaics

**DOI:** 10.1021/acs.jpcc.5c03067

**Published:** 2025-07-30

**Authors:** Christopher J. Blackwell, Tommaso Bianconi, Zachary M. Faitz, Abitha Dhavamani, Martin T. Zanni, Michael S. Arnold

**Affiliations:** †Department of Materials Science and Engineering and ‡Department of Chemistry, 5228 University of Wisconsin−Madison, Madison, Wisconsin 53706, United States

## Abstract

Semiconductor doping has yielded performance enhancements in organic thermoelectrics, light-emitting diodes, transistors, and photovoltaics. In analogous devices using semiconducting single-walled carbon nanotubes (s-SWCNTs), similar doping strategies have been studied for the enhancement of thermoelectrics, light-emitting diodes, and transistors, but virtually no studies have examined the impact of doping on photovoltaic behavior. Here, we investigate chemically p-doped (6,5) s-SWCNTs as light-absorbing donor materials in photovoltaic donor–acceptor heterojunctions with C_60_. Although doping reduces absorption, the photocurrent generated per absorbed photon remains constant up to a dopant density of ∼80 μm^–1^. This unexpected outcome contrasts with the sharp quenching of photoluminescence observed at similar doping levels. We attribute this divergence to the ultrafast electron transfer from s-SWCNTs to C_60_, and we quantitatively describe this system by using a diffusion-limited contact quenching model. We also demonstrate that doping sensitizes s-SWCNTs to generate photocurrent from trions near 1.07 eV. While trion photocurrent is weaker—likely due to reduced diffusivity and a lower driving force for dissociation—it provides a direct route to electrically probe trionic states. These findings establish that doped s-SWCNTs can operate efficiently in photovoltaic devices and serve as a platform for studying trion dynamics in excitonic semiconductors.

## Graphical abstract

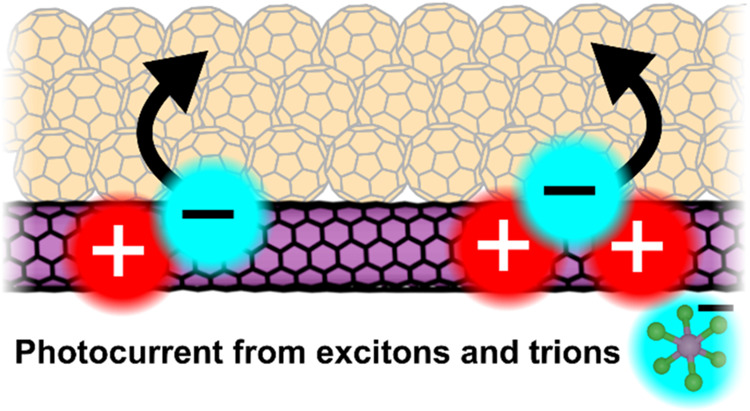

## Introduction

Semiconducting single-walled carbon nanotubes (s-SWCNTs) possess high carrier mobilities, high optical absorptivity, tunable near-infrared bandgaps, and high mechanical and chemical resilience. These properties make them appealing candidates for the primary light absorbers in photovoltaic donor–acceptor heterojunctions.^1^ Devices using polymer-sorted, monodisperse s-SWCNTs as electron donors can convert 85% of absorbed photons into photocurrent (although this high efficiency is currently limited to films <10 nm in thickness).^2,3^

To date, virtually all studies of s-SWCNTs in donor–acceptor heterojunctions have used nearly intrinsic s-SWCNTs without intentional doping.^4^ Doping can change fundamental properties of s-SWCNTs, including the Fermi level,^5^ absorptivity,^6^ conductivity,^5^ and exciton dynamics.^6–8^ These changes can yield meaningful positive or negative impacts on device performance as evidenced in functionally similar organic heterojunctions.^9–16^ Furthermore, the best-performing s-SWCNT devices tend to be processed in halogenated solvents, and it was recently demonstrated that such processing can unintentionally dope s-SWCNTs.^17^ This leaves a potentially significant variable both unexplored and uncontrolled in s-SWCNT heterojunction devices.

P- and n-doping have been studied in isolated s-SWCNTs and in films. Via electrostatic gating, both polarities are attainable. In fact, localized electrostatic doping has been used to form p-n and p-i-n junctions along the length of isolated s-SWCNTs, and these junctions have exhibited photovoltaic behavior.^18,19^

In macroscopic films for energy applications, chemical or charge-transfer doping is a more widely used method. In this technique, a chemical is used to oxidize or reduce the s-SWCNTs. To maintain charge neutrality, counterions then noncovalently adhere to the sidewalls of the s-SWCNTs. Chemically, s-SWCNTs are more susceptible to p-doping than n-doping; even atmospheric oxygen can lightly p-dope s-SWCNTs.^4,20,21^

Charge-transfer-doped s-SWCNTs have been the subject of many spectroscopic studies that have elucidated the roles of both injected charges and counterions.^6,8,22–25^ Perhaps most importantly for s-SWCNT photovoltaics, doping can alter the exciton dynamics. When an exciton diffuses to an injected carrier, it can bind to it to form a trion or charged exciton.^7^ In charge-transfer-doped s-SWCNTs, these trions are localized around counterions, making them generally immobile.^6,8,23^ Counterions may play a similarly important role in the dynamics of neutral excitons; it has been suggested that counterions can form potential wells that localize excitons.^26^ These findings suggest that the impact of doping on photovoltaic device efficiency and behavior, although unexplored, could be significant.

Here, we examine the performance of polymer wrapped (6,5) s-SWCNT–C_60_ photovoltaic heterojunctions over a range of s-SWCNT thicknesses with various levels of p-doping. We measure photocurrent to quantify relative photoinduced electron transfer (PET) efficiency for both excitons and trions. For comparison, we also measure photoluminescence (PL) versus the amount of p-doping in stand-alone s-SWCNT films that are not integrated into a heterojunction. We find that p-doping decreases photocurrent in heterojunctions because injected holes bleach the s-SWCNTs’ *S*_11_ absorption transition and decrease the amount of light absorbed. When normalized by absorbance, however, the photocurrent per absorbed photon remains unchanged for doping densities of up to about 80 μm^–1^. In contrast, the photoluminescence quantum yield of stand-alone films drops by over 90% at an equivalent doping density. We attribute this disparity to the ultrafast electron transfer at the s-SWCNT–C_60_ interface in heterojunctions, which occurs on the order of tens of femtoseconds. We also find that moderate doping can promote a photocurrent at 1.07 eV, corresponding to a peak often ascribed to trion absorption. We estimate that about 7% of photons absorbed at this peak produce a photocurrent. Photocurrent at the trion absorption peak is invariant with reverse bias up to 0.3 V, despite the charged nature of the trion, supporting evidence of the immobile nature of these photoexcitations. These results indicate that p-doped s-SWCNTs are a viable material for use in photovoltaics, albeit with weaker absorption, and that photocurrent from trions is possible, although with a lower quantum efficiency.

## Experimental Methods

### Preparation of s-SWCNTs

A dispersion of s-SWCNTs enriched with the (6,5) chirality is prepared via conjugated polymer extraction with poly [(9,9-dioctylfluorenyl-2,7-diyl)-*alt*-co-(6,60-{2,20-bipyridine})], also known as PFO-BPy.^27,28^ First, 600 mg of PFO-BPy (Montreal Optoelectronics OMI153UV, previously known as American Dye Source ADS153UV) is dispersed in 600 mL of toluene via stirring and heating at 110 °C. 300 mg of CoMoCAT carbon nanotubes (Sigma-Aldrich SG65i) is added and subsequently dispersed via shear force mixing at 10 krpm for 24 h. The s-SWCNT suspension is then centrifuged at 41 krpm for 10 min to remove undispersed material, leaving an enriched dispersion of (6,5) nanotubes in the supernatant. The supernatant is then filtered through a 5 μm filter (Sigma-Aldrich SLSV025LS) and concentrated to 70 mL via rotary evaporation. This polymer-rich dispersion is centrifuged at 50,000*g* for 24 h, precipitating the s-SWCNTs while leaving excess polymer in the supernatant. The pellets are collected and redispersed in toluene with stirring, heating to 60 °C, and bath sonication, followed by another round of centrifugation. They are then redispersed in tetrahydrofuran and centrifuged once more to precipitate the s-SWCNTs while leaving excess polymer in the supernatant. Finally, the pellets are suspended in ortho-dichlorobenzene (ODCB). An absorption spectrum is shown in Figure S1 of the Supporting Information, where we describe the calculations of s-SWCNT and polymer concentration. We find that our stock ink after the above process contains approximately 100 μg/mL (6,5) s-SWCNTs and about 150 μg/mL of PFO-BPy.

### Film Preparation

s-SWCNT films are deposited via drop casting. First, substrates are cleaned via sonication in acetone and isopropanol. They are then treated with ultraviolet light and ozone for 60 min. Small volumes of the stock 100 μg/mL ink are diluted 1–10× and treated with further bath sonication to ensure homogeneity. The diluted inks, typically around 200 μL, are then cast onto substrates and dried over about 48 h. Films are cast in a sealed Petri dish with only a few ventilation holes. This slows the solvent evaporation rate and prevents the formation of a coffee ring. Films intended for devices are cast at a slight angle to achieve a thickness gradient on the substrate. All other films are cast on flat substrates. Once dried, the substrates are immersed in boiling toluene and rinsed with isopropyl alcohol to remove impurities. All samples are made from the same batch of ink, eliminating the concern of batch-to-batch variation in intrinsic doping levels.^29^ Film thicknesses are calculated from atomic force microscopy and absorbance measurements, as shown in Figure S2 of the Supporting Information.

### Absorption Measurements

Light for absorption measurements is generated from a solar simulator consisting of a Xe lamp for ultraviolet and visible light and a quartz tungsten halogen lamp for near-infrared light. The two beams are collinear and combined with a 50/50 beam splitter and directed into a monochromator. Subsequent long pass filters (cutoff wavelengths of 420, 680, and 1090 nm) eliminate unwanted higher-order radiation, and an aperture reduces the beam to approximately 1 mm in diameter. A mechanical chopper modulates the beam, which is then detected by the Newport 818-UV and 818-IR photodetectors. A lock-in amplifier (Stanford Research Systems, Model SR830) is used to measure the chopped signal. We measure natural absorbance as ln­(*I*_reference_/*I*_sample_), where *I* is the current measured by the photodetector.

### s-SWCNT Doping

Doping is performed using the methods demonstrated by Chandra et al. and depicted in Figure 1.^30,31^ Triethyloxonium hexachloroantimonate (OA) is dissolved in dichloroethane (DCE) at 78 °C. s-SWCNT films are submerged in 10 mL of the solution for 5 min before being rinsed in acetone and isopropanol for 2–3 s each. Although OA is known to be an air-stable doping method, all experiments on doped films are nonetheless carried out quickly after doping, typically within 24 h.

**Figure 1:**
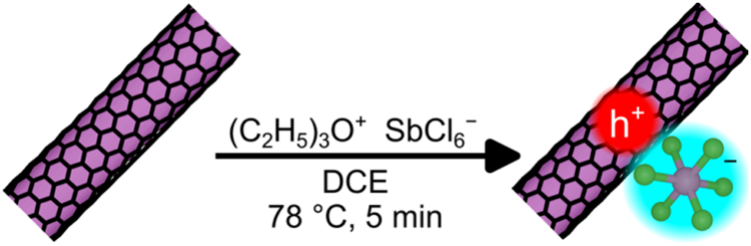
A scheme depicting the doping of s-SWCNTs via OA, a single electron oxidant. In the resulting film, each injected hole is stabilized by a negatively charged hexachloroantimonate anion that remains adsorbed to the s-SWCNT. Wrapping polymer is not pictured for clarity. Not to scale.

To quantify the density of dopants in a film, we use eq 1, where *N*_h_ is the density of dopants in units of nm^–1^ and χ is the fractional change in *S*_11_ oscillator strength after doping, Δ*f*/*f*_0_. Although other relationships can be used to find *N*_h_, we choose eq 1 because it was developed experimentally from (6,5) s-SWCNTs similar to the ones used here.^24^

1 $$N_{h}=0.27\;{\mathrm{nm}}^{-1}\;\chi$$

Since oscillator strength *f* is directly proportional to the area under an absorption peak,^32^ we measure the *S*_11_ absorption of all films before and after doping to quantify Δ*f*/*f*_0_ = ∫*S*_11_ dυ/∫*S*_11,0_ dυ. See Figures S3 and S4 in the Supporting Information for details.

### Photovoltaic Device Fabrication and Measurement

s-SWCNT films are deposited onto indium tin oxide (15 ohms per square, Prazisions Glas & Optik). Control samples are left undoped, while test samples are doped as described above with OA at 0–18 μg/mL. χ is measured using a 1 mm spot size at multiple locations on each film. At these locations, devices are then fabricated by the thermal evaporation of 90 nm of C_60_, 10 nm of bathocuproine, and 120 nm of Ag. The Ag cathodes are circular with a 1 mm diameter and are deposited using a shadow mask.

Photocurrent measurements are taken using the same equipment described above for absorbance measurements. The Newport 818-UV and 818-IR photodetectors have photoresponsivity calibrated from 300 to 1088 and 780 to 1450 nm, respectively. External quantum efficiency (EQE) is found by dividing the device photocurrent by the photon flux measured by calibrated photodetectors.

### Photoluminescence Measurement

A 16 nm s-SWCNT film is deposited onto cleaned quartz as described above. We use quartz rather than borosilicate glass because quartz lacks an emission peak near (6,5) *S*_11_ at 1000 nm. Photoluminescence is performed with a Horiba Nanolog spectrofluorometer. Exciting the (6,5) *S*_22_ peak at 572 nm, emission is detected in the *S*_11_ region from 790 to 1550 nm. The sample is then doped as described above, and χ is measured at multiple points across the surface of the film. The sample is doped a total of five times with OA at 0.5, 1, 5, 10, and 50 μg/mL. After each doping, χ is measured via absorbance, and photoluminescence is measured.

### Transient Absorbance (TA) Measurement

Transient absorbance (TA) is conducted on a 3.5 nm s-SWCNT film on borosilicate glass using a home-built spectrophotometer previously described elsewhere.^33^ We pump and probe the *S*_11_ band from 980 to 1180 nm. 200 mW of 100 kHz output (800 nm) of a Yb:KGW amplifier (PHAROS 1 equipped with iOPA-FW-HP) is split into a pump and probe path using a 70/30 fused silica beam splitter, which is focused on 4 mm YAG crystals to generate the pump and probe pulses (sub-100 fs duration). The probe beam is directed into a motorized delay stage to control the waiting time between the probe and the pump pulses. Transmitted probe light is collected using a Princeton Instruments Acton SP2150 spectrometer and an OMA V InGaAs detector.

## Results and Discussion

### Photoluminescence Measurements

Figure 2a shows absorbance spectra taken from an s-SWCNT film after various degrees of OA doping. Here, we see the characteristic spectral signatures of doped s-SWCNTs: a bleach of the *S*_11_ and an induced absorption peak near 1.07 eV, often identified as the optical formation of a trion.^34^ Recent evidence has challenged this identification and suggested that the 1.07 eV absorption feature could correspond to the formation of a localized exciton^26^ or polaron-dressed exciton.^7^ In this discussion, we adhere to convention and consider the peak to be a signature of trion formation, but we leave open the possibility for alternatives.

**Figure 2:**
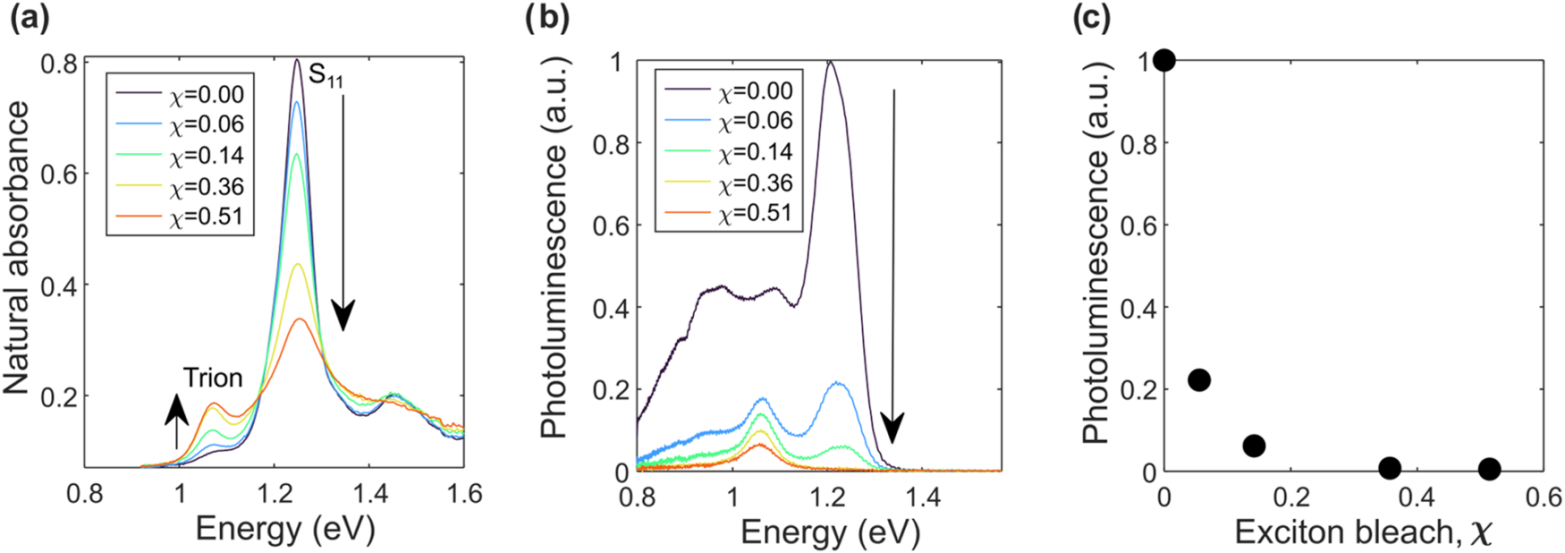
(a) The effects of doping on s-SWCNT thin film absorption spectra in the *S*_11_ region. As χ increases, the *S*_11_ peak flattens, broadens, and slightly blue-shifts. The trion peak intensity increases by χ up to about 0.5. (b) Effects of doping on photoluminescence. Doping severely quenches emission from the *S*_11_ peak and gives rise to a new trion emission feature near 1.07 eV. (c) *S*_11_ PL intensity as a function of χ. Small amounts of doping yield severe reductions in the PL.

Previous studies examined the PL of doped s-SWCNTs in solution and found that PL drops precipitously with doping.^8,35^ In these references, for example, *S*_11_ PL intensity drops by about 90% at χ = 0.2, compared to undoped s-SWCNTs. We reproduce these PL experiments in films. Figure 2b,2c shows the results, which are consistent with the previously reported solution results. A small amount of doping that bleaches the *S*_11_ by only 6% reduces the PL by almost 80%. For χ > 36%, PL at the *S*_11_ is negligible.

The decrease in *S*_11_ PL with doping occurs because excitons diffuse to injected holes and bind to them to form trions rather than radiatively recombining. Note that this trion formation mechanism is generally agreed upon; the only dispute is over whether trions are directly formed optically at 1.07 eV. Via this diffusional process, excitons can efficiently convert to trions.^7^ For this reason, concurrent with the decrease in *S*_11_ PL, we see the emergence of a PL peak at 1.07 eV, corresponding to the radiative decay of trions. Note that the PL emission features seen near 0.9 and 1.1 eV in the undoped s-SWCNT spectrum do not correspond to trion emission: they are substrate-induced emission features commonly found in s-SWCNTs on glass or quartz.^36^

Additionally, we note that these films all absorb approximately the same amount of light upon excitation at the *S*_22_. The *S*_22_ requires more doping than the *S*_11_ before it begins to bleach.^31,35^ Up to about χ = 0.3, the *S*_22_ absorption peak is essentially unchanged. This means that Figure 2c is also a good approximation of the change in photoluminescence quantum yield, PLQY, or photoluminescence per absorbed photon.

### Photocurrent Measurements

To examine the behavior of doped s-SWCNTs in photovoltaic devices, we fabricated typical donor–acceptor heterojunctions, with the structure shown in Figure 3a. Figure 3b shows the corresponding energy diagram. In these devices, a photon is absorbed by the s-SWCNTs to create an exciton. The exciton then diffuses in the s-SWCNT layer until it reaches the heterointerface with C_60_. The driving force for photoinduced electron transfer, Δ*G*_PET_, is related to the binding energy of the exciton, *E*_b_, and the difference in electron affinities between the donor and the acceptor, ΔEA, by eq 2. From 0 meV down to about −130 meV, a more negative Δ*G*_PET_ results in a higher electron transfer yield.^37,38^

2 $$\Delta G_{PET}=E_{b}-\Delta EA$$

**Figure 3:**
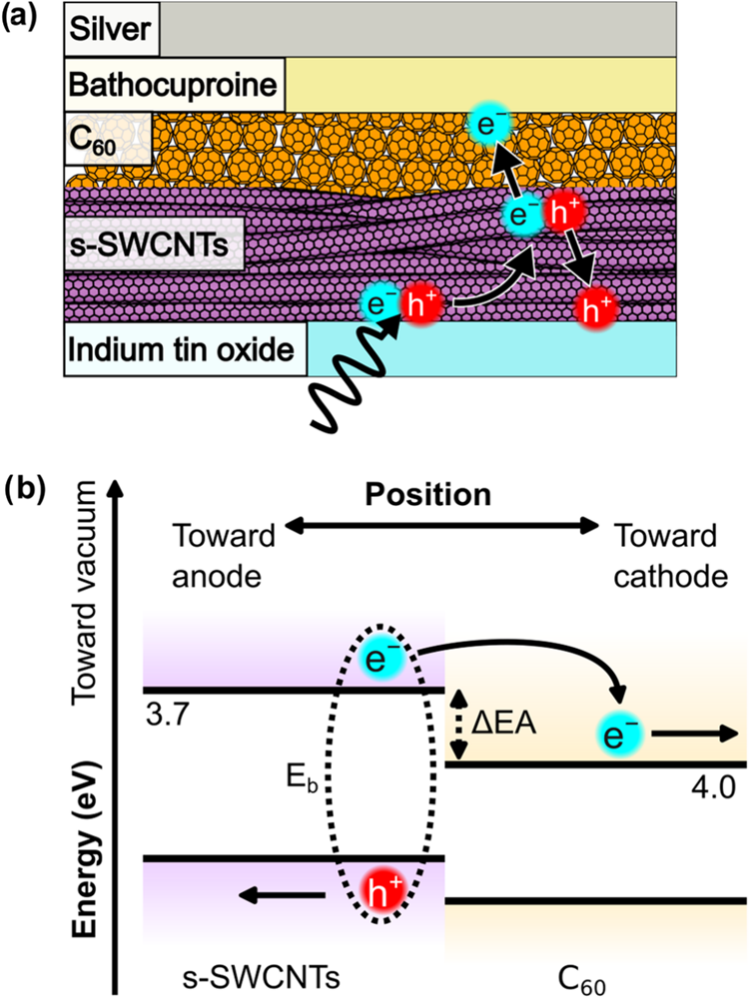
(a) Representative device schematic showing a donor–acceptor heterojunction sandwiched between two electrodes. A photon absorbed by the s-SWCNTs generates an exciton. The exciton then diffuses throughout the s-SWCNT layer until it reaches the heterojunction, where the electron is injected into the C_60_ layer. The electron and hole, now separated, travel to their respective electrodes. (b) Energy versus position diagram of the heterojunction. An exciton is bound by a binding energy *E*_b_. If the difference in electron affinities between the electron donor and acceptor, ΔEA, is greater than *E*_b_, then the exciton can inject an electron into the C_60_. Electron affinities taken from ref 38.

The electron and hole, now unbound, are then collected at the cathode (silver/bathocuproine) and anode (indium tin oxide, or ITO), respectively. This process generates a photocurrent. Here, we quantify the photocurrent at a particular energy as the ratio between the number of electrons generated to the number of incident photons. This is known as external quantum efficiency, or EQE.

In devices made with undoped s-SWCNTs, the EQE changes with the thickness of the s-SWCNT layer. Up to about 8 nm thick, EQE increases with thickness as the thicker films can absorb more photons. When the s-SWCNT layer becomes thicker than 8 nm, EQE begins to decrease with thickness as diffusion losses outpace the enhanced absorption.^39^ See Figure S5 in the Supporting Information. To avoid thickness-related effects in this study, we only characterize devices made with s-SWCNT layers ≤8 nm.

Figure 4a shows the absorbance spectra of three s-SWCNT films on ITO. Each film is approximately 7 nm thick. One film is undoped, while two of them are doped to different degrees. We then fabricate devices from these films and measure the EQE spectra (Figure 4b). As χ increases, the photocurrent at the *S*_11_ peak decreases.

**Figure 4:**
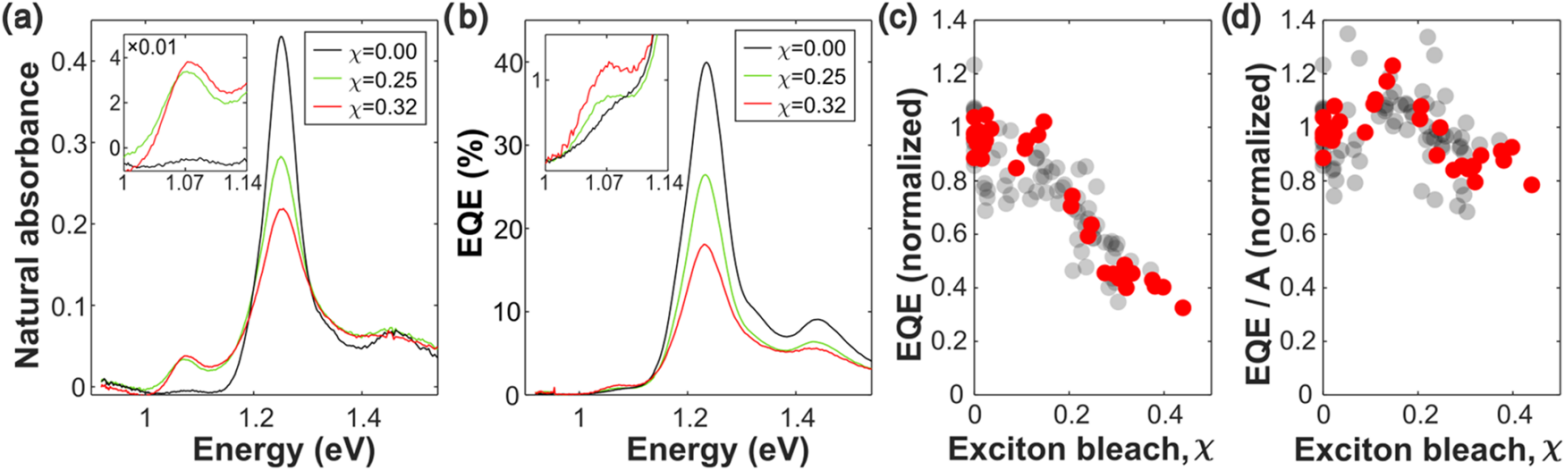
(a) Absorbance spectra of three films with different χ on ITO. Inset: magnified spectra in the trion region. (b) Photocurrent spectra of devices made from the same films with a magnified inset near the trion. (c) *S*_11_ photocurrent as a function of χ, normalized to the photocurrent at χ = 0. Translucent gray markers indicate s-SWCNT films thinner than 6 nm, while red markers indicate s-SWCNT films 7 ± 1 nm thick, comparable to the films and devices shown in parts (a, b). (d) Normalized photocurrent per photon absorbed, found by dividing EQE by *A*. See the Supporting Information. Note the increased noise resulting from thinner films: small variations in photocurrent and absorbance measurements yield large errors after normalization.

In Figure 4c, we plot the normalized peak *S*_11_ photocurrent as a function of χ. We first consider devices with an s-SWCNT layer 7 ± 1 nm thick. These data points are emphasized with opaque red markers. We see that photocurrent decreases linearly with χ. This trend holds for devices with thinner s-SWCNT layers, shown with translucent gray markers.

Photocurrent and photoluminescence are best compared by the signal generated per absorbed photon. To approximate the photocurrent per absorbed photon at the *S*_11_, we divide the photocurrent by absorptance (*A*), the fraction of light absorbed by a film at a particular energy (see Figure S6). The results are shown in Figure 4d. Here, we see that the photocurrent per absorbed photon remains essentially unchanged with doping. This trend holds across all s-SWCNT thicknesses measured (1.7–8.0 nm), as seen from the translucent gray markers.

This starkly contrasts the s-SWCNTs’ PL response with doping. Figure 2c, which is a good approximation of PL per absorbed photon, shows large reductions in the signal after minimal doping. We attribute this behavior to the vastly different time scales on which photoluminescence and photocurrent occur: photoluminescence occurs over picoseconds while photoinduced electron transfer occurs over tens of femtoseconds. This idea is explored more fully in our discussion and modeling below.

### Trion Photocurrent

The insets in Figure 4a,4b show more details around 1.07 eV. As the doping-induced trion peak appears in absorption, we see a much weaker trion peak arise in the photocurrent. For example, the most doped spectrum in Figure 4 (χ = 0.32) exhibits an EQE of 1.18% at 1.07 eV. After subtracting the background caused by the *S*_11_ peak, we find that the trion contribution to the absolute EQE is 0.46%. This is among the highest values we observe in devices with s-SWCNT films thinner than 8 nm.

Trion photocurrent around 1.07 eV has been observed before,^29^ but here we demonstrate that its intensity can be directly controlled via doping, as seen in Figure 5a. For any given thickness of the s-SWCNT layer, a greater bleach (up to χ = 0.4) yields greater trion photocurrent. The photocurrent spectrum with the highest trion photocurrent is shown as the black curve in Figure 5b. This spectrum came from a device with a 16 nm s-SWCNT layer and χ = 0.36. Here, the EQE at 1.07 eV is 1.28%. After subtracting the background caused by the *S*_11_ peak, the trion contribution to the absolute EQE is 0.60%, nearly ten times larger than the previously reported trion EQE.^29^ To better understand the efficiency of the photocurrent from the trion peak, we divide EQE by *A* at the trion peak. By comparing the EQE/*A* at the *S*_11_ and trion peaks (see the Supporting Information), we estimate that 7% (standard deviation 3%) of photons absorbed at the trion peak generate photocurrent.

**Figure 5:**
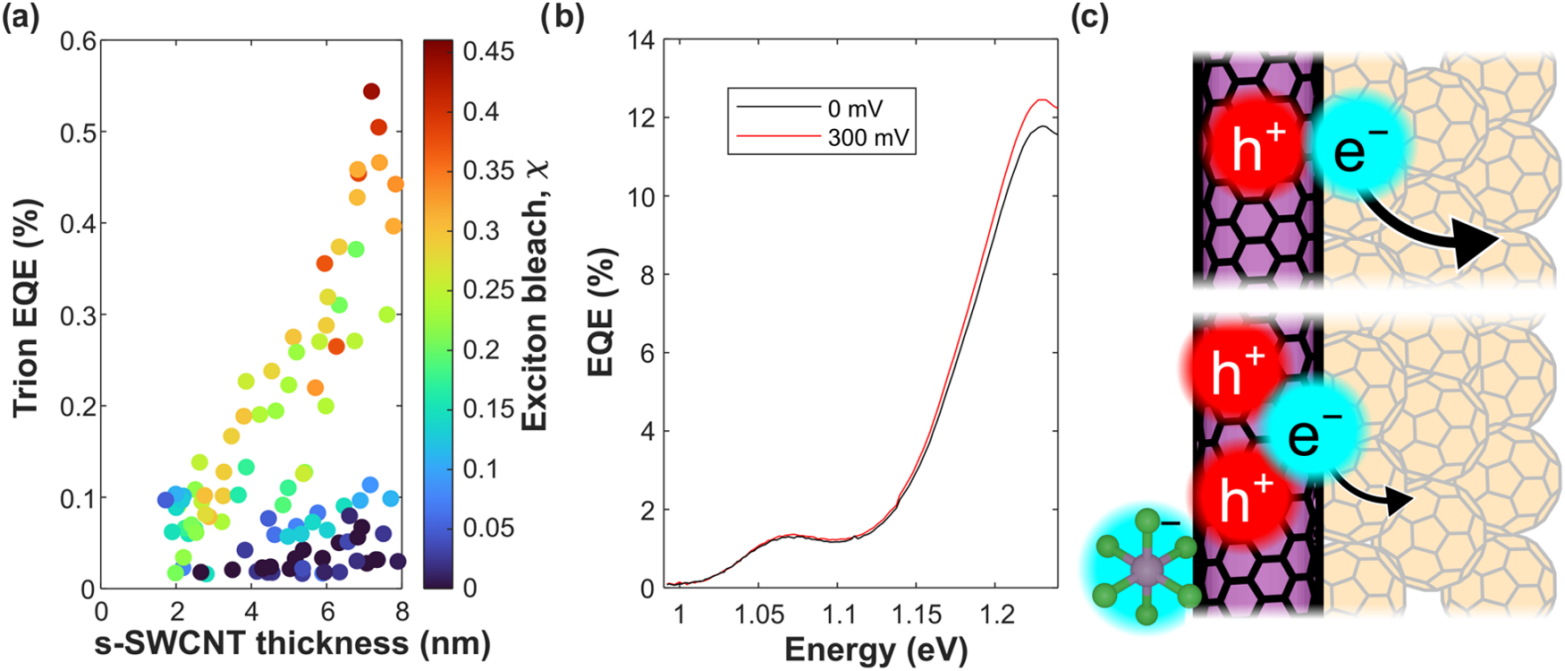
(a) Photocurrent at the trion peak as a function of thickness and χ. At every thickness, more doping yields a greater trion photocurrent. (b) Photocurrent spectrum of a doped device with and without a 300 mV reverse bias. This device produced the highest trion photocurrent that we observed. (c) Illustration of exciton (top) and trion (bottom) dissociation. The extra hole in the trion may lower the efficiency of electron transfer.

We offer three possible explanations for why efficiency is lower at the trion peak than at the *S*_11_. First, trions are effectively immobile compared to excitons in our films. Exciton diffusion is a critical step in photocurrent generation. If an exciton forms at a point buried in the s-SWCNT layer, where it is not in direct contact with C_60_, the exciton must diffuse throughout the s-SWCNTs until it reaches the heterojunction to produce photocurrent. Trions cannot diffuse since they are coulombically locked to a counterion. Any trions formed in a position other than the donor–acceptor interface will not generate photocurrent.

To demonstrate the immobile nature of trions, we apply a reverse bias to a device and observe the trion photocurrent, as seen in Figure 5b. A donor–acceptor heterojunction is effectively a diode. Under a reverse bias, electrons and holes are pulled away from the heterojunction and toward the electrodes. This increases the efficiency of charge collection, which we see as a small increase in the *S*_11_ photocurrent. This reverse bias should also push trions away from the heterojunction, suppressing the photocurrent at the trion peak. We see no meaningful change in EQE at the trion peak with an applied field of 3 × 10^6^ V m^–1^ (300 mV dropped over approximately 100 nm), implying that trions in our films are tightly bound to their counterions.

Second, trions have a lower driving force for dissociation than do excitons. In (6,5) s-SWCNTs, the binding energy of a trion is approximately 160 meV larger than that of an exciton.^34^ As seen in eq 2, a larger binding energy leads to a lower driving force for PET. We would expect this to reduce the yield by over 80% compared to excitons.^37^ Additionally, eq 2 assumes that all charges are separated after electron transfer, an assumption that may not be valid for trions. Electron transfer from a trion to C_60_ leaves two holes in close proximity on the s-SWCNT. This could further decrease Δ*G*_PET_.

Finally, the presence of additional charges at the interface could promote the formation of charge-transfer states that trap charge carriers. In typical s-SWCNT/C_60_ heterojunctions, charge-transfer states are not observed upon exciton dissociation.^29^ When a trion dissociates, however, an electron injected into the C_60_ could be attracted to two holes in the s-SWCNT, as illustrated in Figure 5c. Alternatively, the free hole could be attracted to the counterion.

### Modeling Photocurrent and Photoluminescence

The decrease in PL with χ observed in Figure 2c is well understood. Although the radiative lifetime of excitons in s-SWCNTs is approximately 1 ns, the actual exciton lifetime in solution-processed films is much shorter due to nonradiative quenching.^40,41^ In these films, excitons typically decay within a few picoseconds via intratube diffusion followed by quenching at defects.^42,43^ As the defect density increases, diffusion to quenching sites becomes more efficient, leading to reduced exciton lifetimes and a lower photoluminescence quantum yield. Eckstein et al. demonstrated that dopants quench PL similarly to defects and that the same diffusion-limited contact quenching model can be used to model both.^8^

In contrast, PET at a heterojunction is much faster than PL. The upper bound on τ_PET_, the electron transfer time from s-SWCNT to C_60_, has been measured as 120 fs (instrument limited), and PET in similar systems has been measured at a few tens of femtoseconds.^44,45^ We hypothesize that this ultrafast τ_PET_ promotes electron transfer before excitons can diffuse to a defect or dopant, even at moderate χ. This would explain why the photocurrent per absorbed photon is nearly invariant with χ, as seen in Figure 4d.

To test our hypothesis, we developed a mathematical model to simulate exciton diffusion, photoluminescence, and photocurrent generation in s-SWCNTs, both as stand-alone films and within donor–acceptor heterojunctions. In the model, s-SWCNTs are modified with randomly distributed defects at a density *N*_d_. Dopants are introduced with a variable density, *N*_h_, to represent different doping levels. These defects and dopants partition each s-SWCNT into pristine segments of length *L*_s_, as illustrated in Figure 6a. Because the defects and dopants are placed according to a Poisson point process, there is an exponential distribution of *L*_s_ with a probability density function (PDF) given in eq 3. Here, λ is the average segment length defined in eq 4.

3 $$\mathrm{PDF}\left(L_{s}\right)=\lambda e^{-\lambda /L_{s}}$$

4 $$\lambda =<L_{s}>=1/\left(N_{d}+N_{h}\right)$$

**Figure 6:**
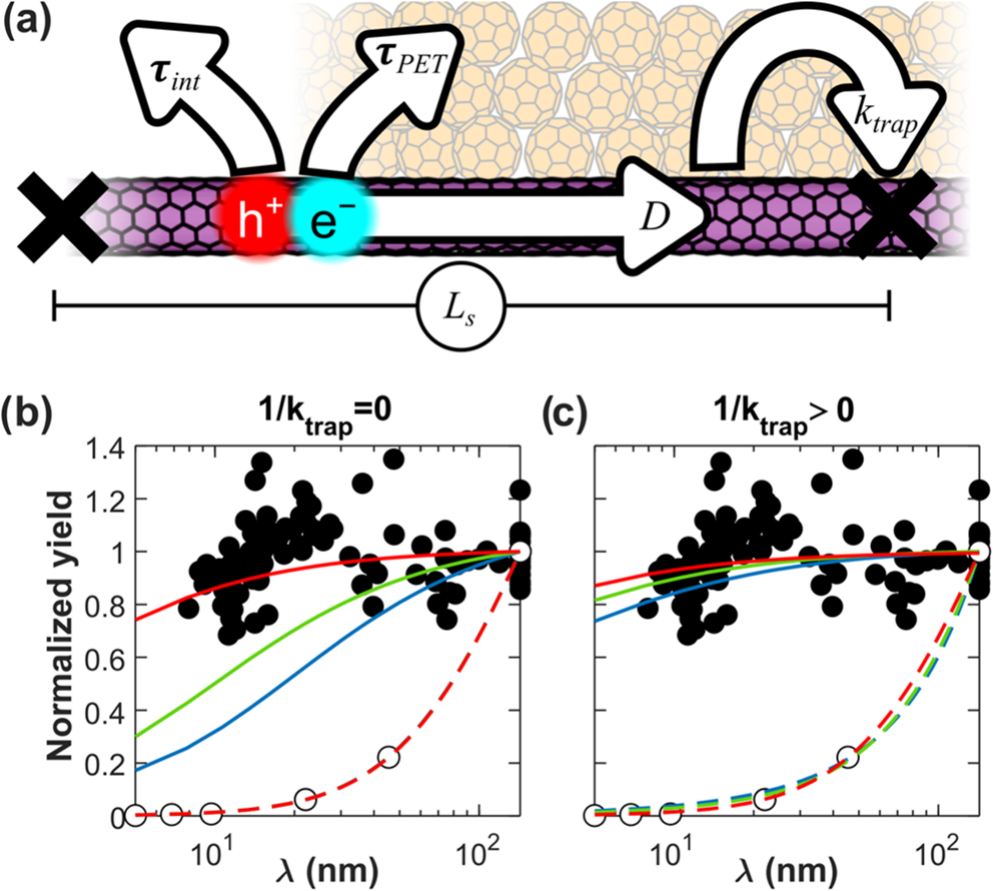
(a) In our model, excitons diffuse along the s-SWCNT until they encounter a defect or dopant. Here, they decay at a rate described by the term *k*_trap_. At any point on the s-SWCNT, excitons can also decay via PL (τ_int_) or, during device simulations, PET to C_60_ (τ_PET_). (b) Comparison of experimental and simulated photocurrent and PL behavior as a function of λ, the spacing between defects or dopants. We assume instant exciton quenching at defects (1/*k*_trap_ = 0). Filled circles show experimental EQE/*A*, while open circles show experimental PL. Solid lines show simulations of photocurrent per absorbed photon in devices, and dashed lines show corresponding simulations of photoluminescence per absorbed photon in films. Blue, green, and red lines indicate simulations in which τ_PET_ = 120, 30, and 1 fs, respectively. As τ_PET_ becomes smaller, the simulations match the data better. (c) Comparison of experimental and simulated data assuming noninstantaneous exciton quenching at defects (1/*k*_trap_ > 0). Colors and markers are the same as in part (b). Here, by allowing *k*_trap_ to vary freely, all three τ_PET_ produce reasonable fits of the photocurrent data. As *k*_trap_ varies, the PL fits shift while retaining a reasonable fit.

The temporal and spatial evolution of excitons in each segment following optical excitation is independently modeled by eq 5. *u*(*x*,*t*) is the exciton density along a segment of length *L*_s_ as a function of position and time. τ_int_ is the intrinsic exciton lifetime, which accounts for radiative and nonradiative recombination processes that are independent of diffusion to defects or electron transfer. Measurements in very long, defect-free s-SWCNTs have shown τ_int_ to be approximately 50–100 ps.^46,47^

5 $$u_{t}=Du_{\mathrm{xx}}-u/\tau_{\mathrm{int}}-u/\tau_{\mathrm{PET}}$$

The full equation is used to model the evolution of excitons in s-SWCNT/C_60_ heterojunctions. When modeling the evolution of excitons in a freestanding s-SWCNT film, like the one used in our photoluminescence experiments, the term containing τ_PET_ is removed, equivalent to setting τ_PET_ = ∞.

At time *t* = 0, we assume a uniform exciton density throughout each segment: *u*(*t* = 0) = 1. The excitons in this model are noninteracting. An initially uniform exciton distribution will, therefore, simulate individual excitons photogenerated randomly along the length of the s-SWCNT segment.

The boundaries of the s-SWCNT segment are *x* = 0 and *x* = *L*_s_. An exciton that has diffused to the boundary of a segment can be quenched or trapped at the rate *k*_trap_. This defines boundary conditions *Du*_*x*_(0,*t*) *= u*(0,*t*)*k*_trap_ and −*Du_x_* (*L*_s_,*t*) = *u*(*L*_s_,*t*)*k*_trap_. A complete solution for *u*(*x*,*t*) can be found in the Supporting Information. After being solved for an arbitrary *L*_s_, *u* is solved for many *L*_s_, and the solutions are averaged together using weights from eq 3 for a particular λ.

To determine the intrinsic defect density in our films, we performed transient absorbance on undoped s-SWCNTs. As seen in Figure S7 of the Supporting Information, 80% of excitons decay within 10 ps, and the decay of the exciton population can be modeled with a stretched exponential. This indicates that decay occurs predominantly through diffusion to defects with very little radiative recombination. We use *u*(*x*,*t*), assuming τ_int_ = τ_PET_ = ∞, to model the exciton population, and we fit *u* to the transient absorbance data. We assume an intratube exciton diffusion constant, *D*, of 8 cm^2^ s^–1^, which lies within the commonly reported range of 1–10 cm^2^ s^–1^.^3^ We find *N*_d_ ≈ 7 μm^–1^, which is in agreement with previous estimates for similar films.^3,27^

For a given χ, we use eq 1 to find *N*_h_ and eq 4 to find λ. For a fixed τ_int_, τ_PET_, and *k*_trap_, we can then model the PLQY for each *L*_s_ by integrating *u*(*x*,*t*)/*τ*_int_ over *x* and *t*; we then average the PLQYs for all *L*_s_ weighted by eq 3. A similar treatment can be used to find the absorbed photon to photocurrent conversion efficiency, or APCE. Since we observe above that approximately 7% of trions generate photocurrent, we also allow 7% of the excitons that decay via *k*_trap_ to contribute to the photocurrent. A more detailed discussion is found in the Supporting Information.

Figure 6b,c shows modeled PLQY and APCE as well as experimental photoluminescence and experimental EQE/*A* as a function of λ (where λ for the experimental data is determined from χ). As previously discussed, normalized PL intensity and EQE/*A* are good approximations of normalized PLQY and APCE. We see that EQE/*A* remains nearly constant with doping up to a dopant density of about 80 μm^–1^. See the Supporting Information (Figure S8) for a discussion of higher dopant densities.

In each figure, we show three different fits of the photoluminescence and photocurrent data. For these fits, we fix *N*_d_ = 7.0 μm^–1^ as determined by transient absorbance, and we assume *D* = 8 cm^2^ s^–1^, which falls in the middle of measurements made for similar films.^3^ In Figure 6b, we assume 1/*k*_trap_ = 0. This implies that excitons instantly form trions upon contact with a dopant and that excitons are instantly quenched upon contact with a defect. We fix τ_PET_ to 120, 30, or 1 fs and let τ_int_ vary as a free parameter. In all three cases, τ_int_ fits to about 14 ps. Best-fit parameters and the root mean squared error (RMSE) are shown in Table 1. Only when τ_PET_ = 1 fs does the model accurately reproduce both the PL and photocurrent data. However, τ_PET_ = 1 fs may be unphysical.

**Table 1:** Parameters Used to Fit *u*(*x*,*t*) to Experimental Data alongside RMSE

τ_PET_ (fs)	τ_int_ (ps)	*k*_trap_ (nm fs^–1^)	RMSE (photocurrent/photoluminescence)
120	13.8	0	0.454/0.002
30	14.2	0	0.315/0.002
1	14.2	0	0.136/0.002
120	1000	81.6	0.143/0.021
30	113	34.4	0.131/0.016
1	16	1.4	0.128/0.003

We address this issue by next considering that trion formation and exciton quenching are not instantaneous, and we allow nonzero 1/*k*_trap_, which we fit as a free parameter alongside τ_int_. The results of these simulations are listed in Figure 6c. All three scenarios produce reasonable fits. Given that recent ultrafast 2D photocurrent spectroscopy data estimate τ_PET_ = 30 fs, τ_int_ and *k*_trap_ of 113 ps and 34.4 nm fs^–1^ are our best estimates of the intrinsic lifetime and trapping rate. Thus, by using a finite but still ultrafast *k*_trap_, much more moderate τ_PET_ values can reproduce experimental observations.

The fits obtained by our model therefore support our hypothesis that ultrafast τ_PET_ outcompetes exciton diffusion to trapping or quenching sites. This result is surprising because s-SWCNTs exhibit very fast exciton diffusion compared to similar materials like organic semiconductors.^3,48^ This suggests that lowering τ_PET_, whether by optimizing ΔEA or morphology,^45^ can be an effective route to increase device performance in any system that is limited by exciton diffusion to defects.

## Conclusions

This work demonstrates that chemically p-doped (6,5) s-SWCNTs retain efficient exciton-to-photocurrent conversion in photovoltaic heterojunctions even at doping levels that strongly suppress photoluminescence. While doping reduces the absorption at the *S*_11_ transition, the photocurrent per absorbed photon remains nearly constant up to a doping density of about 80 μm^–1^. We attribute this to ultrafast photoinduced electron transfer at the s-SWCNT/C_60_ interface, which outpaces exciton diffusion to defects and dopants, and we quantitatively capture this behavior using a diffusion-trapping model. This result implies that mild s-SWCNT doping from exposure to air or halogenated solvents is unlikely to affect photovoltaic device performance as long as s-SWCNTs are in direct contact with C_60_ and τ_PET_ is ultrafast. We also show that moderate doping can increase photocurrent yield at the trion peak to nearly ten times the previously reported values. The efficiency of trion photocurrent remains low overall, likely due to trions’ limited mobility and reduced driving force for dissociation. However, these results suggest that field-controlled trion transport could be used to generate photocurrent in an optimized system with fewer localized counterions and a ΔEA tuned for trion dissociation.

## Supplementary Material



## Notes

Additional experimental details and observations, including atomic force microscopy, transient absorbance, and photocurrent measurements (PDF)
