# Supplementary material for: P‑Doped Carbon Nanotubes as Light-Absorbing Electron Donors in Photovoltaics
Source: J Phys Chem C Nanomater Interfaces. 2025 Jul 30;129(31):13875–83. doi: 10.1021/acs.jpcc.5c03067 (PMC12506631; doi:10.1021/acs.jpcc.5c03067)
Supplement: Supplementary file 1 [file jp5c03067_si_001.pdf]

Supporting Information for P-doped Carbon Nanotubes as Light-absorbing Electron Donors in Photovoltaics

*Christopher J. Blackwell[1], Tommaso Bianconi[2], Zachary M. Faitz[2], Abitha Dhavamani[1], Martin T. Zanni[2], Michael S. Arnold[1]**

[1]Department of Materials Science and Engineering and [2]Department of Chemistry, University of Wisconsin–Madison, Madison, Wisconsin 53706, United States

**Corresponding author: michael.arnold@wisc.edu*

Concentration and purity of sorted (6,5) s-SWCNTs

Figure S1 below shows a normalized natural absorbance spectrum of (6,5)-enriched s-SWCNTs in an ODCB dispersion. Regions containing S_{11}, S_{22}, and S_{33} peaks are highlighted, and relevant peaks are labeled.[1,2] The (6,5) S_{11} and S_{22} appear at 1.23 eV and 2.14 eV, respectively, while their respective exciton-phonon sideband (EPS) peaks appear at 1.43 eV and 2.35 eV. The S_{33} peak is obscured by a large absorption feature around 3.5 eV created by the PFO-BPy. At least two minority chiralities are also present: the (7,5) S_{11} and S_{22} are seen at 1.18 eV and 1.89 eV respectively, while the (9,1) S_{11} is seen at 1.32 eV. An additional absorption feature is seen at 1.08 eV. This could correspond to the S_{11} of another minority chirality, such as (7,6). Alternatively, it could be a trion peak, which would indicate that the s-SWCNTs are weakly unintentionally doped from processing in halogenated solvents.[3]

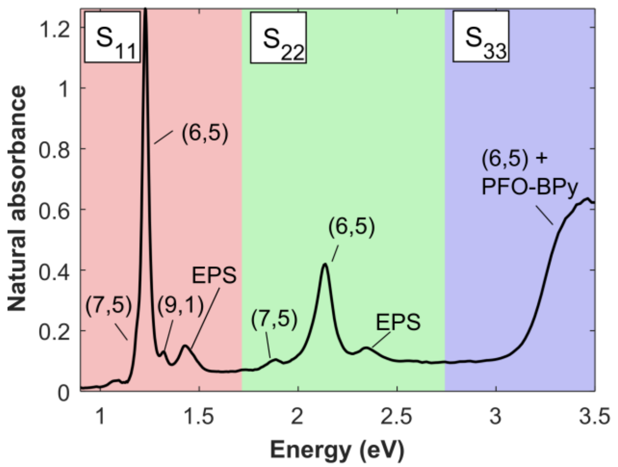

Figure S1: Absorbance spectrum of (6,5)-enriched s-SWCNTs in an ODCB dispersion. This dispersion is diluted 70× from the stock ink, and the spectrum is taken with a 0.875 cm pathlength. From this spectrum, we find that the stock solution was about 112 µg/mL.

The absence of M_{11} absorption peaks in the 2.5-3.0 eV region suggests a high semiconducting purity. Based on the relative intensities of the (6,5), (7,5) and (9,1) S_{11} peaks, we estimate the (6,5) chiral purity to be around 90%. To find the concentration C of (6,5) s-SWCNTs in the dispersion, we use Beer's Law as shown in Equation S1. We use the S_{22} to determine concentration because its area is less susceptible to changes with defects and doping than the S_{11}. $\int S_{22}\,dv$ is the integral of the S_{22} natural absorbance peak, in units of nm^{-1}, as determined by fitting with a Lorentzian peak. For this fitting, we use a linear baseline and include two additional peaks to account for the (7,5) S_{22} and the EPS. l is the path length. $\int \sigma_{22}\,dv$ is the spectrally integrated optical cross section for the (6,5) S_{22}, reported as 5.61×10^{-15} cm per carbon atom.[4]

$$\int S_{22}\ dv = Cl\int \sigma_{22}\ dv \qquad \text{Equation S1}$$

The concentration of PFO-BPy in solution is determined using an absorptivity value of 0.28 $cm^2\ \mu g^{-1}$ found from measured masses of PFO-BPy in solution. Measurements are made at 355 nm.

Finding the thickness of s-SWCNT films

To quantify the thickness of an s-SWCNT film, we again use Equation S1 with Streit's spectrally integrated S_{22} optical cross-sections to find the path length or thickness of the film.[4] Note that Streit's optical cross sections were measured in a solution where s-SWCNTs were randomly oriented in three dimensions. In a thin film, however, the s-SWCNTs are predominantly confined to a two-dimensional plane. s-SWCNTs absorb light most efficiently when the electric field is aligned with the tube's long axis. Integrating the absorption of s-SWCNTs randomly oriented in 2D and 3D reveals that a 2D film absorbs 3/2 more strongly than a 3D dispersion. To account for this, Streit's optical cross sections is scaled up by a factor of 3/2 when calculating film thickness.

For films, $\int S_{22}\, dv$ is fitted from natural absorbance spectra using a pseudo-Voigt function, Equation S2. This linear combination of a Gaussian (f_G) and Lorentzian (f_L) function approximates a Voigt peak with less than 1.2% error while being much simpler computationally.[5] Equations for f_G and f_L are given as Equations S3 and S4. η is a mixing parameter, while I determines the intensity of the peak. x_0 is the center position of the peak. γ_G and γ_L are parameters describing the width of the Gaussian and Lorentzian components.

$$f_{pV}(x) = I\big((1-\eta)f_G + \eta f_L\big) \qquad \text{Equation S2}$$

$$f_G(x,\gamma_G) = (1/(\pi^{1/2}\gamma_G))\exp\,(-(x-x_0)^2/\gamma_G^2) \qquad \text{Equation S3}$$

$$f_L(x,\gamma_L) = (1/(\pi\gamma_L))(1+(x-x_0)^2/\gamma_L^2)^{-1} \qquad \text{Equation S4}$$

In this form, a pseudo-Voigt peak can be fit with five parameters: I, η, x_0, γ_G and γ_L. With our pseudo-Voigt fit defined, we first prepare our natural absorbance spectra for fitting by subtracting the substrate baseline (quartz or indium tin oxide). We then subtract a local linear baseline around the S_{22}. We concurrently fit the (7,5) S_{22}, and EPS to further isolate the (6,5) S_{22} and obtain an accurate integral.

To solve for l in Equation S1, we must define C, the concentration or density of the film. We assume a film density of 1.2 g cm^{-3}, consistent with that of an s-SWCNT network.[6] Given a polymer-to-nanotube mass ratio of 3:2, the nanotube fraction constitutes 40% of the total mass. Accordingly, we estimate the effective s-SWCNT density within the film to be 0.48 g cm^{-3}.

Figure S2a shows an AFM image of an s-SWCNT film on glass alongside a corresponding absorbance spectrum. Via absorbance, we measure this film to be 11.5 nm thick (standard deviation 1.02 nm). Figure S2b shows an AFM image of the same film after some of the s-SWCNTs are scratched away. The corresponding height profile is also given. Via AFM, we find that this film is 10.0 nm (1.04 nm) thick, closely agreeing with the absorbance measurement.

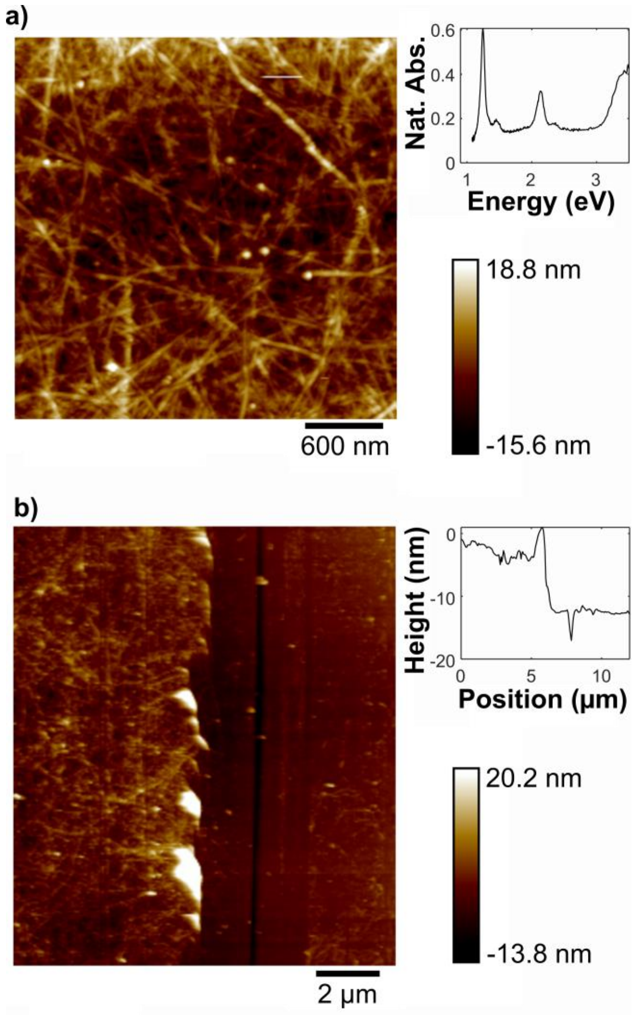

Figure S2: a) AFM showing individual s-SWCNTs in a film (average roughness 4 nm) alongside its absorbance spectrum. b) AFM showing a scratch in the s-SWCNT film. The graph in the upper right shows the average height profile of every line in the image (corrected for tilt).

Extracting χ from absorbance spectra

We use the S_{11} bleach, χ, to quantify doping intensity in films. χ is defined by Equation S5, where f is the oscillator strength of the S_{11} transition.

$$\chi = 1 - f/f_0 \qquad \text{Equation S5}$$

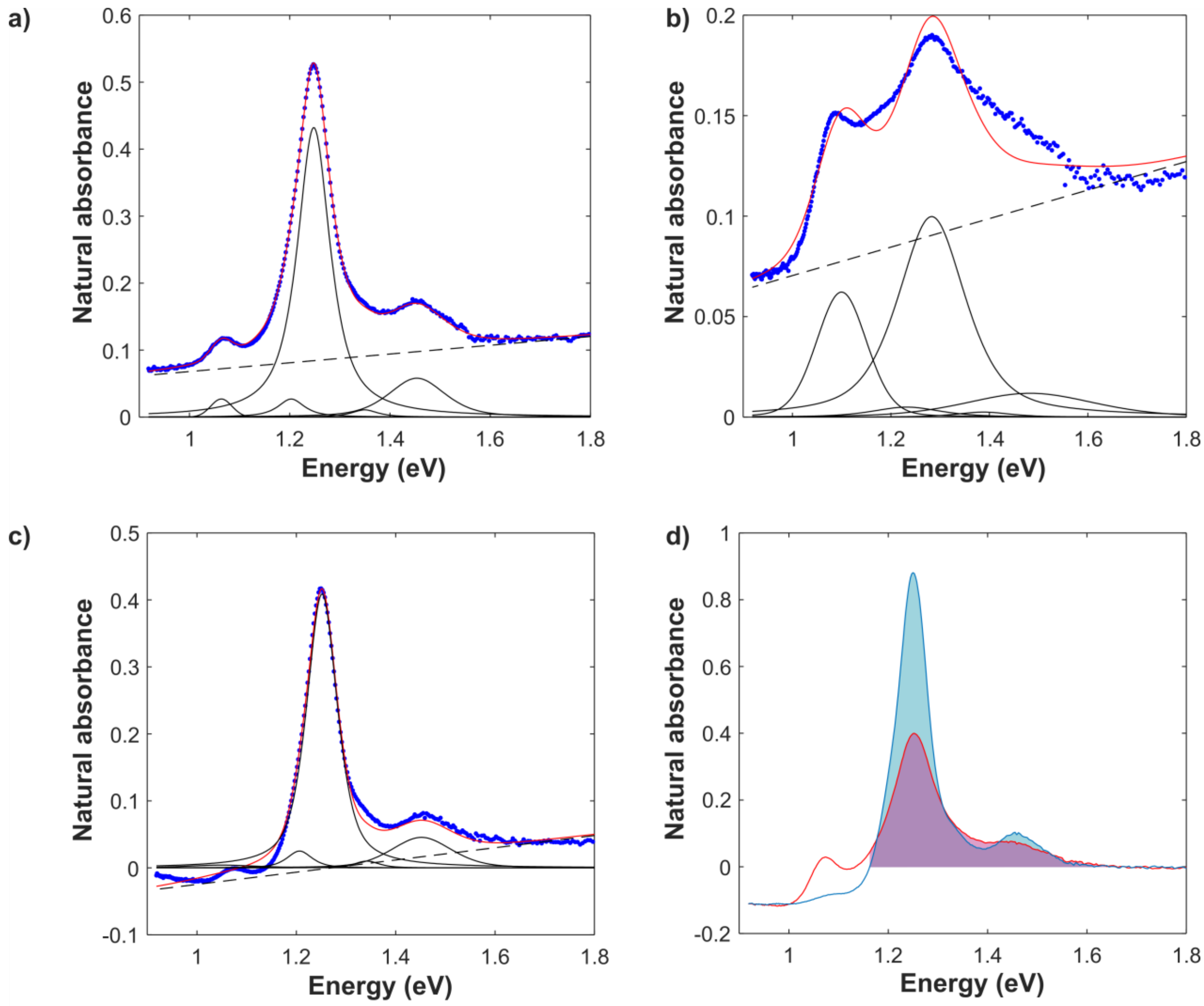

Figure S3: a) Pseudo-Voigt fitting of the S_{11} peak of a lightly doped s-SWCNT film on quartz. b) Fitting of a heavily doped film on quartz. Deviations from standard peak shapes become apparent. c) Fitting of a lightly doped s-SWCNT film on ITO. The negative baseline near 1 eV adds complexity. d) Undoped (blue) and moderately doped (red) s-SWCNT absorption spectra on ITO. The shaded regions, including the entirety of the EPS, are included in integral used to find χ.

f/f_0 is proportional to $\int S_{11}\, d\upsilon / \int S_{11,0}\, d\upsilon$, the ratio of the area under the S_{11} absorption peak before and after doping. In undoped and lightly doped films on quartz, the S_{11} absorption peak can be easily fit to a pseudo-Voigt profile as shown in Figure S3a. However, in moderately and heavily doped films, the S_{11} peak broadens and combines with the EPS and trion peaks, deviating from a conventional lineshape. An example is seen in Figure S3b. The peaks in the S_{11} region become broad and overlap, and it becomes difficult to assign oscillator strength to the (6,5) S_{11}.

Further complications arise when fitting absorption spectra on indium tin oxide. ITO is reflective in the near-infrared region. s-SWCNTs form an antireflective coating on ITO leading to a negative absorption baseline around 1 eV, as seen in Figure S3c. This nontrivial baseline changes with s-SWCNT thickness.

To accurately find $\int S_{11}\, d\upsilon$ with a consistent method regardless of substrate or χ, we adopt the practices of Eckstein et al. who approximated $\int S_{11}\, d\upsilon$ as the area under the entire absorption curve in the S_{11} region, including the EPS.[7] We further restrict ourselves to only positive absorption, not considering absorption near 1 eV which could be negative on ITO. We demonstrate this in Figure S3d, which shows the areas considered for $\int S_{11}\, d\upsilon / \int S_{11,0}\, d\upsilon$. For spectra on both ITO and quartz, we subtract a linear baseline defined by the 1.6 – 1.8 eV region. We then find the area under the spectrum between 1.6 eV and the energy at which the undoped absorption curve becomes negative, typically about 1.15 eV.

We also note that χ depends on both the concentration of OA used to dope a film as well as the film's thickness. Figure S4 shows χ measured for several s-SWCNT films of different thicknesses. All of these films are doped with 4 µg/mL of OA in DCE, yet the thinner films exhibit a smaller χ. We suspect that the acetone and isopropanol rinsing steps from the doping procedure more effectively remove dopants from thinner films.

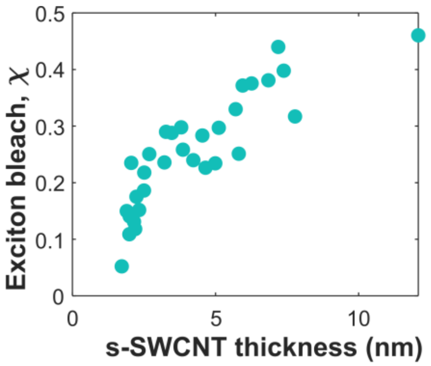

Figure S4: A given concentration of OA in the doping solution can field different χ depending on the s-SWCNT film thickness. Here, an OA concentration of 4 µg/mL is shown to yield χ ranging from 0.05 for a 2 nm film to 0.45 for a 12 nm film.

Photocurrent measurements

Figure S5a shows S_{11} *EQE* as a function of thickness for devices made with undoped s-SWCNTs (χ=0). Closed markers indicate samples that did not undergo any doping step. Open markers indicate devices made from films that experienced the doping process at 0 µg/mL of OA. We see no evidence that exposure to DCE during the doping process affects device performance. We then find a continuous function for EQE by fitting these data to the empirical form of Equation S6 where l is thickness, m= 0.08685, a= 0.09979, and b= 5.337.

$$EQE_{\chi=0} = ml - a * ln(1 + exp(l - b)) \qquad \text{Equation S6}$$

We then assess if the χ of a doped s-SWCNT film changes after thermal evaporation of C_{60}, the first step in the device-making process after doping. During thermal evaporation, the s-SWCNT film is kept at high vacuum (approximately 1e-6 Torr). Figure S5b shows absorbance spectra of an s-SWCNT film before doping, after doping, and after deposition of 15 nm of C_{60}. We see no significant change in χ after thermal evaporation, indicating that doped s-SWCNT films remain doped when fabricated into devices.

To normalize the data shown in Figure 4c of the main text, we divide the S_{11} *EQE* by $EQE_{\chi=0}$ from Equation S6. In Figure 4d of the main text, we plot EQE/A divided by $EQE_{\chi=0}/A_{\chi=0}$. Here, $A_{\chi=0}$ and A are the absorptance measured from a film before and after doping respectively.

An alternative view of the photocurrent data is shown in Figure S6. Figure S6a shows the absorptance of the S_{11} peak of all samples before and after doping. Figure S6b shows peak S_{11} photocurrent as a function of the thickness and χ for all devices measured. For any fixed thickness, *EQE* decreases with χ. And as discussed in the main text, *EQE* for a fixed χ tends to increase with thickness until about 8 nm, at which point *EQE* decreases with thickness. This critical thickness is slightly larger than in previous studies, which reported about 5 nm. Differences in defect density and polymer content between studies could slightly alter the optimal film thickness.[8–10] Additionally, most previous studies examined films made via doctor blading which could exhibit slightly different morphologies compared to the drop casted films examined here.

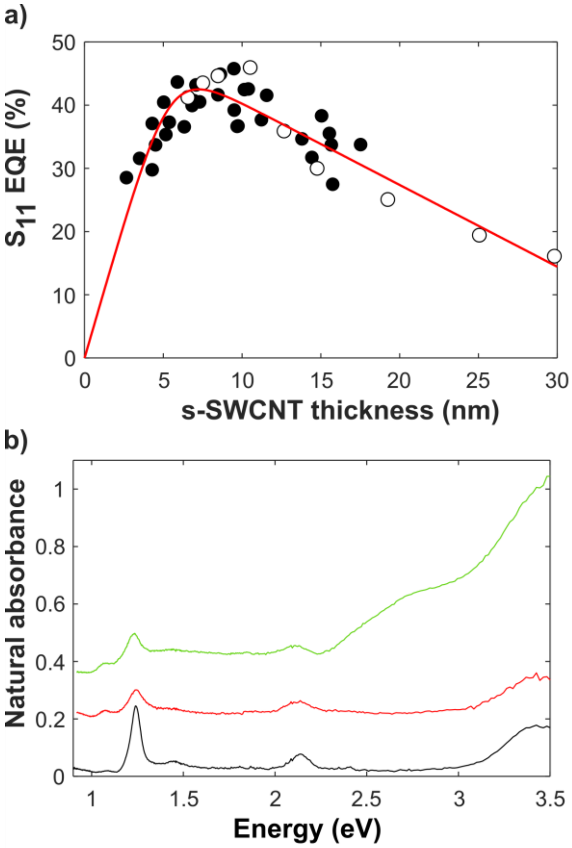

Figure S5: a) EQE as a function of thickness for undoped s-SWCNTs. Open markers indicate samples that are exposed to the doping solvents without OA (in other words, doped at 0 µg/mL). The red line shows the best fit for this data (R^2 = 0.78). b) Absorbance spectra demonstrating that doping persists through C_{60} deposition. The black bottom curve shows the absorbance spectrum of a 2.5 nm s-SWCNT film on ITO. The red middle curve shows the same film after doping to χ=0.4. The green top curve shows the same film after depositing 15 nm of C_{60}. Each curve is offset by 0.2 units.

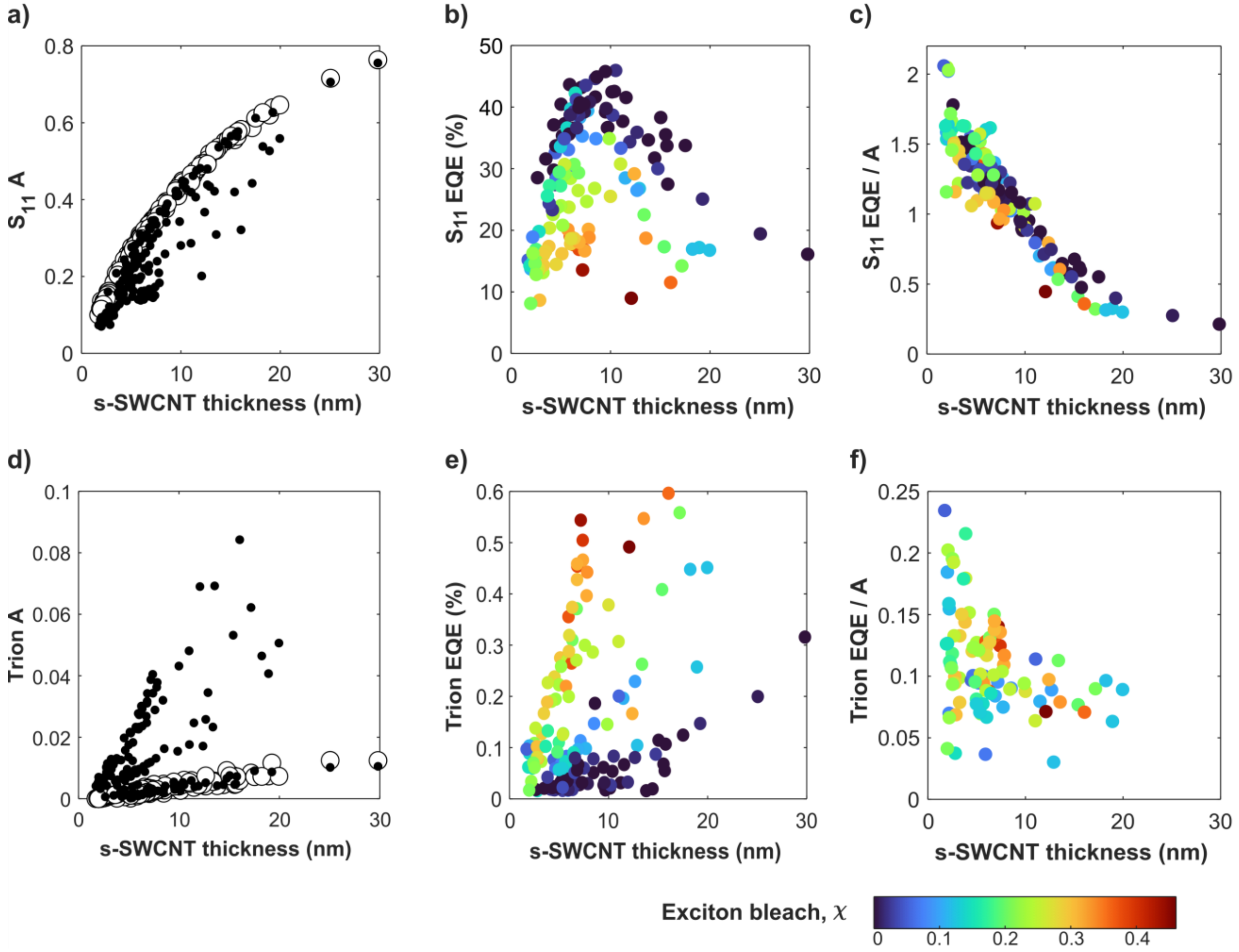

Figure S6: a,d) Absorptance of s-SWCNT films at the a) S_{11} and d) trion peak. Pre-doping (χ=0) absorptance is shown in as the larger open markers, while doped absorptance is shown as the smaller closed markers. b,e) Photocurrent from devices made from these s-SWCNT films at the b) S_{11} and e) trion peak. c,f) Photocurrent divided by absorptance at the c) S_{11} and f) trion peak. Colorbar corresponds to b),c),e), and f). *EQE/A* at χ=0 are not shown in f) because the small denominator drastically exaggerates the photocurrent.

In Figure S6c, we plot *EQE/A* at the S_{11} for all devices. While *EQE/A* is useful for comparing APCE across devices, it should not be taken as an absolute measure of APCE. This is because the absorptance, *A*, was calculated from absorbance spectra of s-SWCNT films prior to device fabrication. Once C_{60}, bathocuproine, and Ag are added, optical interference effects can significantly alter how much light is absorbed by the s-SWCNT layer. These effects can either enhance or suppress absorption relative to the standalone film. For example, some devices in Figure S6c show *EQE/A* > 1.0, indicating that interference has increased absorption in the s-SWCNT layer beyond what was measured before device assembly. Despite this, the optical interference effects should be similar across our devices due to the consistent layer structure and narrow thickness range. Therefore, *EQE/A* remains a valid metric for comparing device performance at a single wavelength. Focusing on devices with a single s-SWCNT thickness in Figure S6c, we observe no dependence of *EQE/A* on χ.

We examine trion photocurrent with similar caution. Figure S6d shows absorptance at the trion peak before and after doping. Figure S6e then shows *EQE* at the trion peak as a function of χ and thickness for all samples. Figure S6f shows *EQE/A* at the trion peak. We only show devices with $\chi>0.05$ because as the trion absorption goes to zero, *EQE/A* becomes erroneously large.

From 0–8 nm, the average *EQE/A* at the trion peak is 0.12 (standard deviation 0.04), compared to 1.4 (0.25) at the S_{11}. As a first approximation, we assume that optical interference effects at 1.07 eV are similar to those at the S_{11}.[11] Based on this, we estimate that trion photocurrent is about 8% as efficient as S_{11} photocurrent. Prior studies have shown that 85% of photons absorbed at the S_{11} generate photocurrent.[12] Applying this factor, we estimate that approximately 7% (±3%) of photons absorbed at the trion peak contribute to photocurrent.

Solving Equation 5 from the main text

Here, we demonstrate how to solve the differential equation presented in the main text for a single s-SWCNT segment length, L_s. Then, we assemble a weighted average of many L_s weighted by Eq. 3. We begin with the equation:

$$u_t = Du_{xx} - u/\tau_{PET} - u/\tau_{int} \quad \text{Equation S7}$$

For simplicity, we can combine τ_{int} and τ_{PET} into a single quantity and simplify Eq S7

$$\tau = 1/(1/\tau_{PET} + 1/\tau_{int}) \quad \text{Equation S8}$$

yielding

$$u_t = Du_{xx} - u/\tau \quad \text{Equation S9}$$

The initial condition is defined as

$$u(x,0) = u_0(x) = 1 \quad \text{Equation S10}$$

We use the Robin boundary conditions

$$Du_x(0,t) = u(0,t)k_{trap} \quad \text{Equation S11}$$

$$-Du_x(L_s,t) = u(L_s,t)k_{trap} \quad \text{Equation S12}$$

to represent boundaries at which the exciton population decays at a nonzero finite rate. Here, k_{trap} has the unintuitive units of nm fs^{-1}. k_{trap} encompasses the rate at which excitons are trapped and the characteristic exciton-trap interaction length. Equivalently, $u \times k_{trap}$ equals a 1D flux.

To begin solving for u, we first use a change of variables to further simplify the equation. We define

$$v(x,t) = u(x,t)e^{t/\tau} \quad \text{Equation S13}$$

which simplifies Eq S9 to

$$v_t = Dv_{xx} \quad \text{Equation S14}$$

This also changes the boundary conditions to

$$Dv_x(0,t) = v(0,t)k_{trap} \quad \text{Equation S15}$$

$$-Dv_x(L_s,t) = v(L_s,t)k_{trap} \quad \text{Equation S16}$$

Next, we assume that time and position are independent, and we use a separation of variables

$$v(x,t) = X(x)T(t) \quad \text{Equation S17}$$

which yields

$$X(x)T'(t) = DT(t)X''(x) \quad \text{Equation S18}$$

Rearranging Eq S18, we find that a function of t is equal to a function of x, implying that both functions must be equal to a constant. Here, we define the constant as $-k^2$, a definition which will facilitate our spatial solution.

$$\frac{T'(t)}{DT(t)} = \frac{X''(x)}{X(x)} = -k^2 \quad \text{Equation S19}$$

The form

$$T(t) = e^{-Dk^2 t} \quad \text{Equation S20}$$

satisfies the temporal component of Eq S19. To solve the spatial component, we try *X* of the form

$$X(x) = Acos(kx) + Bsin(kx) \quad \text{Equation S21}$$

If we define

$$\beta = \frac{k_{trap}}{D} \quad \text{Equation S22}$$

then Eq S19 combined with the boundary condition Eq S15 yields

$$X'(0) = \beta X(0) \quad \text{Equation S23}$$

Combining Eq S21 and Eq S23, we find that

$$X(0) = A \quad \text{Equation S24}$$

$$X'(0) = Bk \quad \text{Equation S25}$$

Combining these with Eq S23, we find

$$B = \frac{\beta}{k} A \quad \text{Equation S26}$$

which yields

$$X(x) = A\left[\cos(kx) + \frac{\beta}{k}\sin(kx)\right] \quad \text{Equation S27}$$

Applying the other boundary condition Eq S16 to the spatial solution in Eq S27 yields

$$-Aksin(kL_s) + A\beta\cos(kL_s) = -\beta A\left[\cos(kL_s) + \frac{\beta}{k}\sin(kL_s)\right] \quad \text{Equation S28}$$

This is an eigenvalue problem; for known k_{trap}, D, and L_s, there are n values of k that satisfy Eq S28. Equivalently, we have the eigenvalue condition

$$(\beta^2 - k_n^2)\sin(k_n L_s) + 2\beta k_n \cos(k_n L_s) = 0 \quad \text{Equation S29}$$

with eigenfunction

$$X_n(x) = \cos(k_n x) + \beta/k_n \sin(k_n x) \quad \text{Equation S30}$$

To solve for X, we use a Fourier series:

$$X(x) = \sum_{n=0}^{\infty} A_n X_n(x) \quad \text{Equation S31}$$

Before solving for A_n, we will consider that each spatial solution X_n will have a corresponding temporal solution T_n:

$$T_n(t) = e^{-Dk_n^2 t} \quad \text{Equation S32}$$

Combining these, we find

$$v(x,t) = X(x)T(t) = \sum_{n=0}^{\infty} A_n X_n(x) T_n(t) = \sum_{n=0}^{\infty} A_n e^{-Dk_n^2 t} X_n(x) \qquad \text{Equation S33}$$

Removing our change of variables yields the solution

$$u(x,t) = e^{-t/\tau} \sum_{n=0}^{\infty} A_n e^{-Dk_n^2 t} \left[\cos(k_n x) + \frac{\beta}{k_n} \sin(k_n x)\right] \qquad \text{Equation S34}$$

If simulating photoluminescence in a film, the equation does not change except that τ is replaced with τ_{int}. If simulating photocurrent in a device, we use Eq S8 to reincorporate τ_{int} and τ_{PET}:

$$u(x,t) = e^{-t/\tau_{int}} \, e^{-t/\tau_{PET}} \sum_{n=0}^{\infty} A_n e^{-Dk_n^2 t} \left[\cos(k_n x) + \frac{\beta}{k_n} \sin(k_n x)\right] \qquad \text{Equation S35}$$

We then solve for the Fourier coefficients

$$A_n = \frac{\int_0^{L_S} u_0(x)\left[\cos(k_n x) + \frac{\beta}{k_n}\sin(k_n x)\right] dx}{\int_0^{L_S} \left[\cos(k_n x) + \frac{\beta}{k_n}\sin(k_n x)\right]^2 dx} \qquad \text{Equation S36}$$

Computationally, we solve for *u* out to n=1000. This solution is for a single L_s. For a given λ, $u(x,t)$ is found for many L_s larger and smaller than λ, and they are then averaged together with weights determined by Eq 3 of the main text.

Using transient absorbance to find N_d

Exciton decay in solution-processed, monodisperse s-SWCNT films is dominated by nonradiative quenching following diffusion to a defect. In these systems, the exciton population decays according to a stretched exponential with a characteristic decay time τ_0, as shown in Equation S37.[13,14] We observe this behavior using transient absorbance spectroscopy, which tracks changes in optical density (OD) over time. We pump and probe the entire S_{11} region from 980-1180 nm which, in undoped s-SWCNTs, almost exclusively excites S_{11} excitons.

$$\Delta OD(t) = OD_0 \exp\left(-\left(\frac{t}{\tau_0}\right)^{1/2}\right) \qquad \text{Equation S37}$$

The resulting decay curve is shown in Figure S7a. Plotting the curve on altered axes (Figure S7b), we see a linear decay characteristic of the stretched exponential form. This confirms that the exciton lifetime is diffusion limited.

To extract the defect density from this spectrum, we use model described by Equation S35 with τ_{int}=τ_{PET}=1/k_{trap}=0 and D=8 cm^2 s^{-1}. We let λ vary as a free parameter, and we simulate 1000 L_s logarithmically spaced around each λ. We only fit after the first 250 fs because the decay does not follow the stretched exponential form at very short time delays. The best fit, shown in Figure S7a, corresponds to λ=143 nm, or N_d≈7 μm^{-1}.

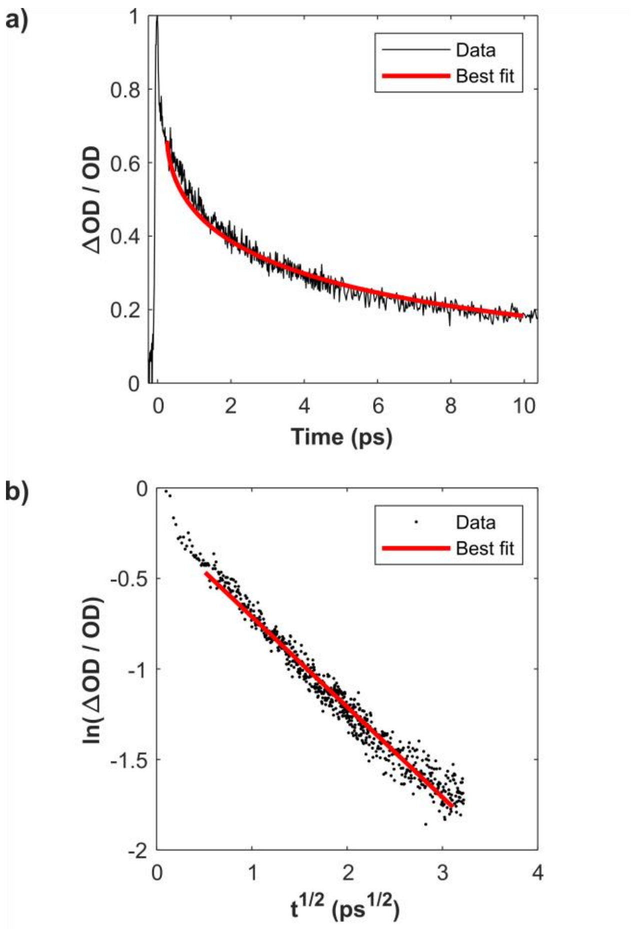

Figure S7: a) Transient absorbance spectrum of an undoped s-SWCNT film pumping and probing at the S_{11}. The best fit from the diffusion model is shown in red. b) The same spectrum shown with altered axes. Extracting the slope of the decay, we find τ_0=4.0 ps.

Using Equation 5 to fit photocurrent and photoluminescence

For a given L_s, we define the simulated APCE as

$$APCE = \int_{x=0}^{x=L_S} \int_{t=0}^{t=\infty} \frac{u(x,t)}{\tau_{PET}} dt dx \qquad \text{Equation S38}$$

using u defined in Eq S35. For a given λ, we calculate APCE for 100 L_s spaced around λ, weight each APCE according the Equation 3, and average them together.

We use a similar process for PLQY. To model PLQY, we find Q_{int}, the fraction of excitons that decay via τ_{int} using Eq S34 and Equation S39. τ_{int} describes radiative and nonradiative decay processes that do not change with doping, so it does not yield an absolute value of PLQY. The ratio between Q_{int} for two different λ, however, is the same as the ratio of PLQYs.

$$Q_{int} = \int_{x=0}^{x=L_S} \int_{t=0}^{t=\infty} \frac{u(x,t)}{\tau_{int}} \qquad \text{Equation S39}$$

Observations at higher doping densities

Our study focuses on lightly and moderately doped s-SWCNTs (χ<0.5). In this regime, the S_{11} mostly retains its peak shape making quantitative analysis straightforward. The S_{11} also retains most of its oscillator strength, making it useful in light-absorbing applications. We focus less on the high doping regime (χ≥0.5) because changes in the S_{11} peak shape obfuscate quantitative analysis and because applications for highly doped s-SWCNTs are less obvious. Still, we present an example of a highly-doped s-SWCNT device for qualitative analysis.

Figure S8 shows absorbance and EQE corresponding to 4 nm s-SWCNT films with χ=0 and χ=0.74 (dopant density of 200 μm^{-1}). At χ=0.74, absorptance at the S_{11} drops by 82%, and EQE drops by nearly 96% compared to χ=0. $EQE_{\chi=0.74}/A_{\chi=0.74}$ divided by $EQE_{\chi=0}/A_{\chi=0}$ is only 0.24. This behavior does not conform to any of our simulations, and it suggests a regime change at high doping densities. At room temperature, it is believed that injected hole wavefunctions overlap at a dopant density of 200 μm^{-1}. Furthermore, this overlap is believed to cause spin entanglement between injected holes.[15] Further research is needed to elucidate what exactly this means for the behavior of excitons at a heterojunction, but the onset of spin entanglement could have dramatic implications for device performance.

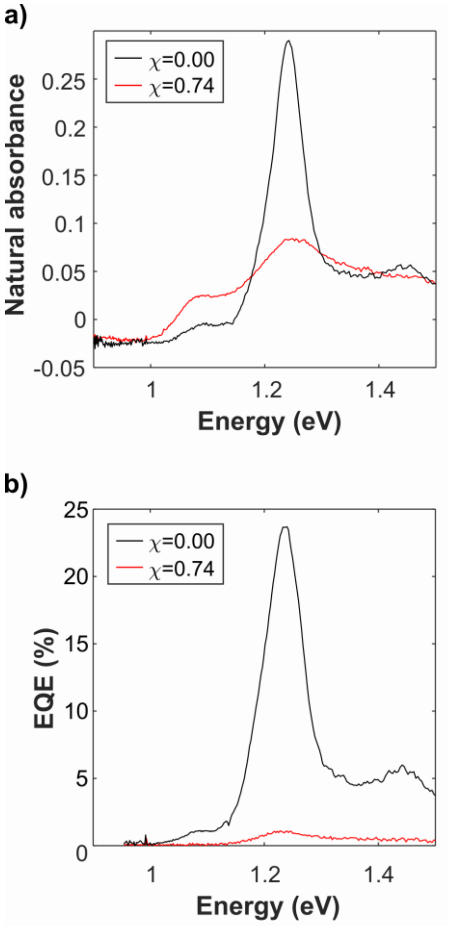

Figure S8: a) Absorbance spectra of two 4 nm thick s-SWCNT films on ITO with different doping conditions. b) Corresponding photocurrent for devices fabricated from these films.

References

(1) Pfohl, M.; Tune, D. D.; Graf, A.; Zaumseil, J.; Krupke, R.; Flavel, B. S. Fitting Single-Walled Carbon Nanotube Optical Spectra. *ACS Omega* **2017**, *2* (3), 1163–1171. https://doi.org/10.1021/acsomega.6b00468.

(2) Weisman, R. B.; Bachilo, S. M. Dependence of Optical Transition Energies on Structure for Single-Walled Carbon Nanotubes in Aqueous Suspension: An Empirical Kataura Plot. *Nano Lett.* **2003**, *3* (9), 1235–1238. https://doi.org/10.1021/nl034428i.

(3) Taborowska, P.; Stando, G.; Sahlman, M.; Krzywiecki, M.; Lundström, M.; Janas, D. Doping of Carbon Nanotubes by Halogenated Solvents. *Sci. Rep.* **2022**, *12* (1), 7004. https://doi.org/10.1038/s41598-022-11162-3.

(4) Streit, J. K.; Bachilo, S. M.; Ghosh, S.; Lin, C.-W.; Weisman, R. B. Directly Measured Optical Absorption Cross Sections for Structure-Selected Single-Walled Carbon Nanotubes. *Nano Lett.* **2014**, *14* (3), 1530–1536. https://doi.org/10.1021/nl404791y.

(5) Ida, T.; Ando, M.; Toraya, H. Extended Pseudo-Voigt Function for Approximating the Voigt Profile. *J. Appl. Crystallogr.* **2000**, *33* (6), 1311–1316. https://doi.org/10.1107/S0021889800010219.

(6) Wu, M.-Y.; Jacobberger, R. M.; Arnold, M. S. Design Length Scales for Carbon Nanotube Photoabsorber Based Photovoltaic Materials and Devices. *J. Appl. Phys.* **2013**, *113* (20), 204504. https://doi.org/10.1063/1.4805597.

(7) Eckstein, K. H. Linear and Nonlinear Spectroscopy of Doped Carbon Nanotubes. Dissertation zur Erlangung des naturwissenschaftlichen Doktorgrades, Julius-Maximilians-Universität Würzburg, Würzburg, 2019.

(8) Wang, J.; Shea, M. J.; Flach, J. T.; McDonough, T. J.; Way, A. J.; Zanni, M. T.; Arnold, M. S. Role of Defects as Exciton Quenching Sites in Carbon Nanotube Photovoltaics. *J. Phys. Chem. C* **2017**, *121* (15), 8310–8318. https://doi.org/10.1021/acs.jpcc.7b01005.

(9) Shea, M. J.; Wang, J.; Flach, J. T.; Zanni, M. T.; Arnold, M. S. Less Severe Processing Improves Carbon Nanotube Photovoltaic Performance. *APL Mater.* **2018**, *6* (5), 056104. https://doi.org/10.1063/1.5026853.

(10) Bindl, D. J.; Shea, M. J.; Arnold, M. S. Enhancing Extraction of Photogenerated Excitons from Semiconducting Carbon Nanotube Films as Photocurrent. *Chem. Phys.* **2013**, *413*, 29–34. https://doi.org/10.1016/j.chemphys.2012.08.001.

(11) Shea, M. J.; Arnold, M. S. 1% Solar Cells Derived from Ultrathin Carbon Nanotube Photoabsorbing Films. *Appl. Phys. Lett.* **2013**, *102* (24), 243101. https://doi.org/10.1063/1.4811359.

(12) Bindl, D. J.; Arnold, M. S. Efficient Exciton Relaxation and Charge Generation in Nearly Monochiral (7,5) Carbon Nanotube/C_{60} Thin-Film Photovoltaics. *J. Phys. Chem. C* **2013**, *117* (5), 2390–2395. https://doi.org/10.1021/jp310983y.

(13) Hertel, T.; Himmelein, S.; Ackermann, T.; Stich, D.; Crochet, J. Diffusion Limited Photoluminescence Quantum Yields in 1-D Semiconductors: Single-Wall Carbon Nanotubes. *ACS Nano* **2010**, *4* (12), 7161–7168. https://doi.org/10.1021/nn101612b.

(14) Harrah, D. M.; Swan, A. K. The Role of Length and Defects on Optical Quantum Efficiency and Exciton Decay Dynamics in Single-Walled Carbon Nanotubes. *ACS Nano* **2011**, *5* (1), 647–655. https://doi.org/10.1021/nn1031214.

(15) Sperlich, A.; Eckstein, K. H.; Oberndorfer, F.; Sturdza, B. K.; Auth, M.; Dyakonov, V.; Mitric, R.; Hertel, T. Onset of Spin Entanglement in Doped Carbon Nanotubes Studied by EPR. *J. Chem. Phys.* **2024**, *160* (23), 234702. https://doi.org/10.1063/5.0207502.
